# Supplementary material for: Chondroitin-Sulfate-A-Coated Magnetite Nanoparticles: Synthesis, Characterization and Testing to Predict Their Colloidal Behavior in Biological Milieu
Source: Int J Mol Sci. 2019 Aug 22;20(17):4096. doi: 10.3390/ijms20174096 (PMC6747333; doi:10.3390/ijms20174096)
Supplement: Supplementary file 1 [file ijms-20-04096-s001.pdf]

Supplementary Materials

1. Characterization of CSA dissociation

The pristine Na_2CSA and the H-ion exchanged CSA (H-CSA) were subjected to thermal analysis (Fig. S1). The Na_2CSA and the H-CSA have a weight loss of ~12% and ~20%, respectively, up to 170°C caused by the evaporation of adsorbed water. The degree of sulfonation of Na_2CSA was calculated as 0.95, from its ash content (Na_2SO_4) and the amount of evaporated water.

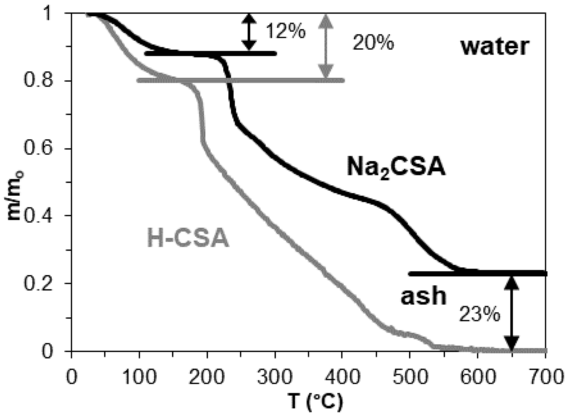

Figure S1. TG curves of the pristine Na_2CSA and the synthesized H-CSA samples.

We intended to characterize the dissociation degree of CSA polyelectrolyte. The primary result of potentiometric acid-base titrations is the sum of net proton consumption in all probable processes such as hydrolysis, for example, occurring in parallel with the protonation or deprotonation of the functional groups (e.g., $-COOH \leftrightarrow -COO^- + H^+$ or $-SO_3H \leftrightarrow -SO_3^- + H^+$).

The primary net proton consumptions of Na_2CSA (Fig. S2) are in the positive range under pH ~6, i.e., protons are consumed by some alkaline species present in the solution of untreated CSA. We have experienced similar effect of base contamination in some non-monocationic forms of clays [1,2]. To get a well-defined initial state, we had to prepare a hydrogen-form of CSA (see in Materials section). The net proton consumption curves of H-CSA (Fig. S2) are in good agreement with the expected tendencies, i.e., the negative values even at the lowest pH indicate a small proton deficit on CSA due to dissociation of –COOH, which significantly rises up to pH ~7.

The net proton consumption curves show a characteristic ionic strength-dependence, i.e., the degree of dissociation of functional groups is influenced by not only the pH but also the salt concentration. The pH-dependent dissociation curves of polyelectrolytes are typically different from that of their monomers [3]. The functional groups are close to each other in a polyelectrolyte, so in the dissociated form, they generate an electrostatic field along the chain of the polyanion. The dissociation of the next proton from the already negatively charged chain is electrostatically hindered. Simultaneously, the ions of the indifferent electrolyte distribute in the local field; thus, they have a charge-screening effect proportional to the ionic strength. This explains the characteristic ionic strength-dependence of polyelectrolytes, i.e., at fixed pH, the degree of dissociation increases with the increasing ionic strength.

The value of the net proton consumption of H-CSA at the fully deprotonated range is ~ –2.05 mmol/g (Fig. S2), which corresponds to one mole of deprotonated functional group on one mole of repeating unit considering its molar mass (459 g/mol). During the hydrogen-form preparation, the pH of Na_2CSA solution was set to ~1. The strongly acidic sulfate groups can be fully deprotonated even at this pH [40,41], so only the protonation/deprotonation of the $-COOH/-COO^-$ groups could be measured in the course of the titration. The evaluation of the Na_2CSA's measurement data became possible by comparing the net proton consumption curves of Na_2CSA and H-CSA, and assuming the

hydrolysis of the sodium salt of CSA: $Na_2CSA + H_2O \leftrightarrow NaHCSA + Na^+ + OH^-$. On the basis of this, Na_2CSA releases ~0.95 mol OH^- per one mole of repeating units.

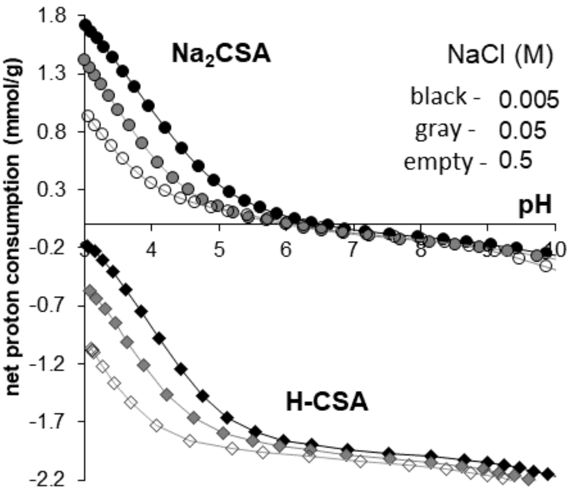

Figure S2. Net proton consumption curves of Na_2CSA and H-CSA obtained from potentiometric acid-base titrations at different NaCl concentrations.

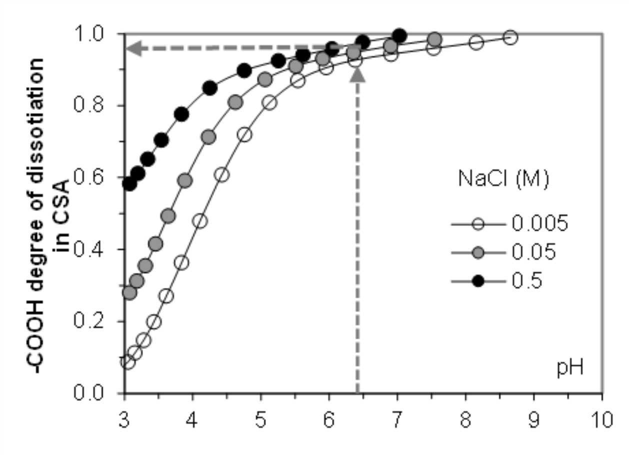

Figure S3. pH- and ionic strength-dependent charging of CSA, the dashed lines indicate the degree of dissociation at pH ~6.3 and 10 mM NaCl, respectively.

The net proton consumption values of H-CSA can be converted to the degree of dissociation of –COOH groups. The pH- and ionic strength-dependent charging of CSA (independent of the forms Na-CSA or H-CSA) is seen in Fig. S3. With the increase in salt concentration, the same degree of dissociation can be achieved at gradually lower pH values due to charge screening of NaCl. The dashed lines point to the dissociation degree of CSA (proportional to the charge state) at the pH and ionic strength of the CSA@MNP preparation (pH ~6.3 and 10 mM NaCl), where the CSA polyions are strongly negatively charged, since the degrees of dissociation are ~1.0 for $–SO_3H$ (fully dissociated in the pH range of the experiments) and ~0.95 for –COOH groups. According to the degree of sulfonation (0.95 as determined by TG) and degrees of dissociations, one mole of repeating units of CSA contains 0.95 moles of fully deprotonated $–SO_3^-$, 0.05 moles of –COOH and 0.95 moles of $–COO^-$ groups at pH~6.3 and 10 mM NaCl. The total amount of negative charges at the pH and ionic strength of the experiments is 1.9 mol/mol of repeating units.

2. Characterization of colloidal stability

The critical coagulation concentration (CCC) of a naked and three CSA-coated MNP dispersions was determined by DLS method. The average hydrodynamic diameter (ZAve) was measured at 25±0.1 °C as a function of time at different salt concentrations (c) (see the kinetic curves of three systems in Figure S4). The initial slope of the kinetic curves is proportional to the coagulation rate (k_{actual}) at the given salt concentration (see the slope values in the right columns in Figure S4). The coagulation rate is small at sufficiently small salt concentrations and increases with increasing salt concentration until reaching a maximum value and remains constant in the fast coagulation regime (k_{fast}). The stability ratio (W) can be calculated as the ratio of the initial slopes belonging to the fast and slow coagulation for each salt concentration, i.e., $W_{actual}=k_{fast}/k_{actual}$.

The salt tolerance of the samples is given as the critical coagulation concentration (CCC) obtained from the stability ratio ($\log_{10}W$) vs. electrolyte concentration ($\log_{10}$ c) functions (see Fig. 6.) as the intersection point of the fast (W_{actual}~1) and slow (W_{actual}>1) coagulation regimes (marked with red arrows in Fig.6.).

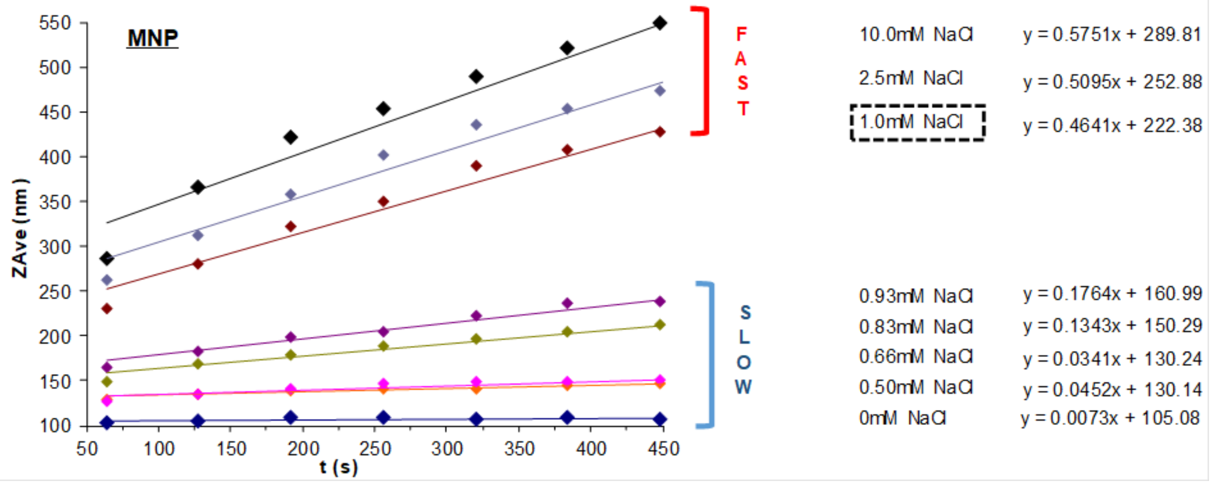

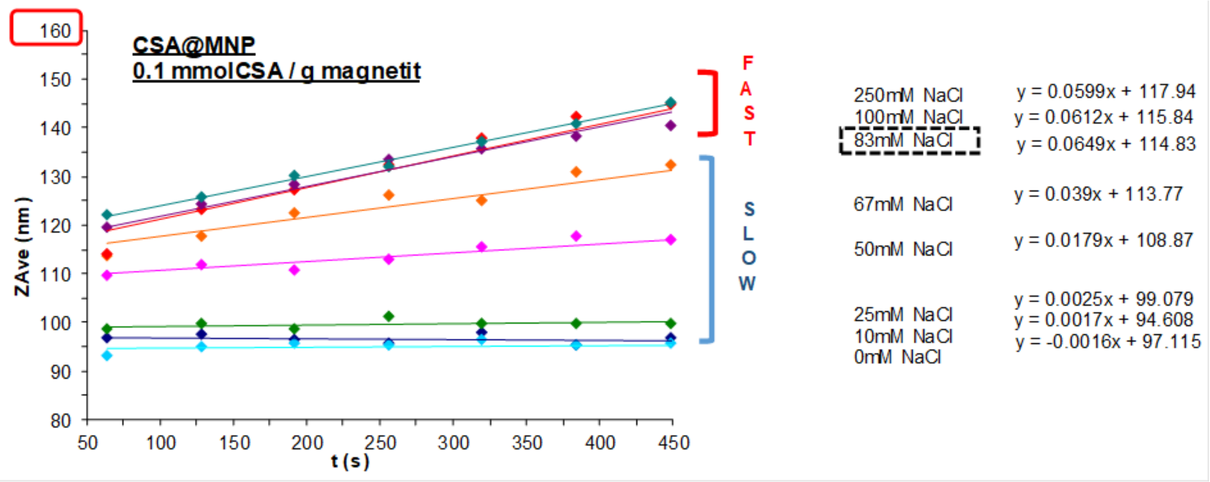

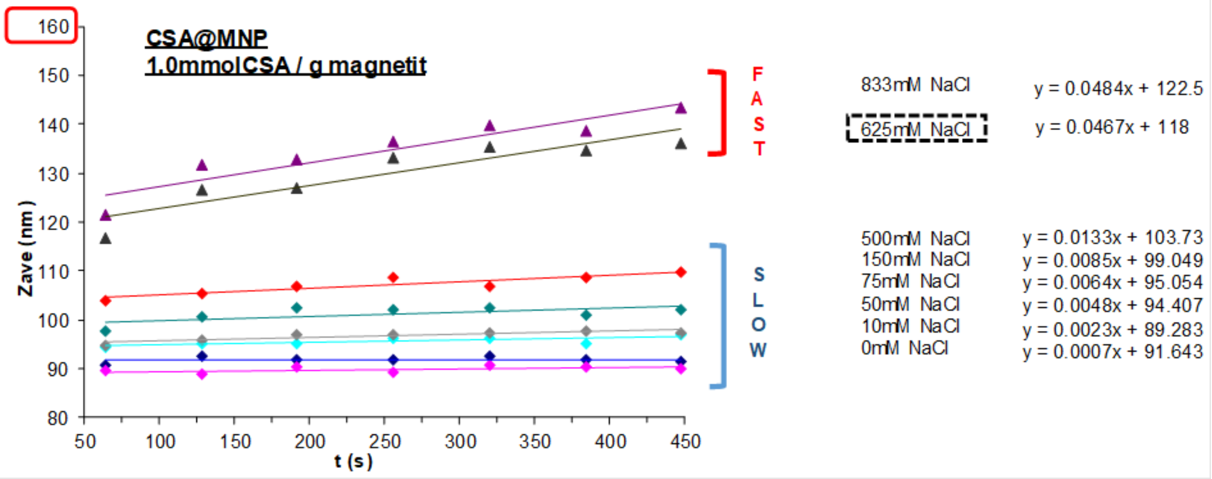

Figure S4. Coagulation kinetic curves of naked (top), partly (middle) and fully (bottom) CSA-coated MNP dispersions at pH~6.3, measured at 25°C. The fast and slow coagulation regimes are indicated. The results of the linear fitting of the kinetic curves are shown in the right column.

Materials and Methods

1. Materials

The hydrogen-form of CSA (H-CSA) was prepared by the following method: the pH of the Na_2CSA solution was adjusted to pH ~1, then this solution was dialyzed against ultra-pure water, and finally the product was lyophilized. The H-form of CSA was tested by thermogravimetry and used for the potentiometric acid-base titration only.

2. Thermogravimetry

Thermogravimetric analyses of Na_2CSA and H-CSA were carried out in a MOM Q-1500 D Derivatograph, in the temperature range of 25–1000 °C at 5 °C/min heating rate.

3. Acid-Base Titrations

Potentiometric acid-base titrations were performed according to the procedure described previously [1,4] to characterize the pH- and ionic strength-dependent charging of CSA by GIMET1 automatic home-made titration system. The background electrolyte NaCl (0.005, 0.05 and 0.5 M) was indifferent with no specific interactions of its ions with CSA. The data points of the titration results express equilibrium states; the equilibrium criterion of ΔpH/min < 0.012 was used.

References

1 Szekeres, M.; Tombácz, E. Surface charge characterization of metal oxides by potentiometric acid-base titration, revisited theory and experiment. *Colloid. Surface. A* **2012**, *414*, 302–313.

2 Tombácz, E.; Szekeres, M. Interfacial acid-base reactions of aluminium oxide dispersed in aqueous electrolyte solutions. 1. potentiometric study on the effect of impurity and dissolution of solid phase. *Langmuir* **2001**, *17*, 1411–1419.

3 Borkovec, M.; Jönsson, B.; Koper, G. J. M. Ionization processes and proton binding in polytropic systems: small molecules, proteins, interfaces and polyelectrolytes. In *Surface and Colloid Science*, Vol. 16.; Matijevic, E., Ed; Kluwer Academic: New York, 2001; pp 99–339.

4 Tóth, I. Y.; Illés, E.; Bauer, R. A.; Nesztor, D.; Szekeres, M.; Zupkó, I.; Tombácz, E. Designed polyelectrolyte shell on magnetite nanocore for dilution-resistant biocompatible magnetic fluids. *Langmuir* **2012**, *28*, 16638–16646.
